# The LOV-domain blue-light receptor LreA of the fungus *Alternaria alternata* binds predominantly FAD as chromophore and acts as a light and temperature sensor

**DOI:** 10.1016/j.jbc.2024.107238

**Published:** 2024-03-28

**Authors:** Lars Schuhmacher, Steffen Heck, Michael Pitz, Elena Mathey, Tilman Lamparter, Alexander Blumhofer, Kai Leister, Reinhard Fischer

**Affiliations:** 1 Department of Microbiology, Institute for Applied Biosciences, Karlsruhe Institute of Technology (KIT) - South Campus, Karlsruhe, Germany; 2 Joseph Kölreuter Institute for Plant Research, Karlsruhe Institute of Technology (KIT) - South Campus, Karlsruhe, Germany

**Keywords:** flavin chromophore, Alternaria, fungi, WC-1, *Neurospora crassa*, circadian rhythm, temperature sensing, signal transduction, blue-light sensing, FAD

## Abstract

Light and temperature sensing are important features of many organisms. Light may provide energy but may also be used by non-photosynthetic organisms for orientation in the environment. Recent evidence suggests that plant and fungal phytochrome and plant phototropin serve dual functions as light and temperature sensors. Here we characterized the fungal LOV-domain blue-light receptor LreA of *Alternaria alternata* and show that it predominantly contains FAD as chromophore. Blue-light illumination induced ROS production followed by protein agglomeration *in vitro*. *In vivo* ROS may control LreA activity. LreA acts as a blue-light photoreceptor but also triggers temperature-shift-induced gene expression. Both responses required the conserved amino acid cysteine 421. We therefore propose that temperature mimics the photoresponse, which could be the ancient function of the chromoprotein. Temperature-dependent gene expression control with LreA was distinct from the response with phytochrome suggesting fine-tuned, photoreceptor-specific gene regulation.

Light receptors are chromoproteins comprised of a protein and an organic light-absorbing molecule called chromophore. In general, photoreceptors are activated by the absorption of a photon with a specific wavelength by the chromophore. The absorbed energy leads to signal transduction within the photoreceptor and ultimately to conformational changes and activation of the protein. Light receptors for blue, green, and red light have evolved in bacteria, fungi, plants, and animals (1, 2). In addition to receptors for the visual part of the light spectrum, some organisms have also developed receptors for detecting UV light. While fungi do not have such receptors, plants, among others, can detect ultraviolet light with the photoreceptor UVR-8 (3, 4).

While photoreceptors for the visual part of the light spectrum use a special chromophore for light sensing, UV-light receptors use aromatic amino acids (4, 5). Phytochrome, the red-light receptor of plants, bacteria, and fungi, uses a linear tetrapyrrole, and green-light receptors like rhodopsin use retinal (6, 7, 8). Blue light perception is one of the most diversified forms of light perception due to the different classes of blue light receptors. Depending on different chromophore binding domains, three classes can be distinguished: blue-light receptors, which use a LOV (**L**ight-**O**xygen-**V**oltage) domain for chromophore binding, the ones which use a BLUF (**B**lue-**L**ight-**U**sing-**F**AD) domain, and the CPF (**c**ryptochrome **p**hotolyase **f**amily) (9, 10, 11, 12). While BLUF proteins and the CP family use FAD (**f**lavin **a**denine **d**inucleotide), LOV-domain blue light receptors from plants, bacteria, and fungi may harbor RBF (**r**i**b**o**f**lavin), FMN (**f**lavin **m**ono**n**ucleotide), or FAD as chromophore. While the blue-light receptor phototropin from plants is generally associated with FMN, fungal blue-light receptors show a more diverse spectrum of chromophores (13, 14, 15, 16, 17). Blue-light receptors are also distinguished from red and green-light receptors by the binding of the chromophore. In their dark-adapted state, the chromophore of LOV domain receptors is non-covalently bound in a chromophore-binding pocket. When exposed to light, a covalent bond forms between the flavin and the protein. The establishment of the covalent bond and the photocycle is in almost every case of short duration. During completion of the photocycle, the covalent bond is cleaved and the dark-adapted state regenerates (14, 18).

Interestingly, filamentous fungi may use all three classes of photoreceptors to sense blue, green, and red light (10, 19). One of the best-studied blue-light photoreceptors is the *Neurospora crassa* white-collar protein WC-1. Almost all photoresponses in this fungus depend on that protein. It forms a heterodimer with WC-2, called the white-collar complex, and binds to the promoters of light-regulated genes. The WCC plays a central role in regulating circadian rhythms in *N*. *crassa*. The regulation of circadian rhythms is influenced, among other things, by light and temperature stimuli and is dependent on a feedback loop of the WCC with vivid (VVD) and frequency (FRQ) at the cellular level (20, 21, 22). WC-1 contains FAD as a chromophore and combines photoreceptors with transcriptional activator functions (23, 24). In comparison, *Aspergillus nidulans* uses mainly the red-light sensor phytochrome for most photoresponses, although it also contains WC-1 and WC-2 orthologues (25, 26). *Alternaria alternata* also contains both, phytochrome and WC orthologues but in addition a functional rhodopsin (27). The phytochrome of both fungi is also able to sense temperature (28). Such a dual function of phytochrome had been reported before for plants (29, 30). However, whereas the temperature-sensing function of phytochrome in plants was light-dependent, fungal phytochrome acts as temperature sensor already in the dark. Besides red-light receptors, also blue-light receptors like the phototropin from plants or Vivid from *N*. *crassa* were shown to be temperature sensitive (31, 32, 33).

Here we characterized the WC-1 orthologue, LreA, from *A. alternata* and show that it contains FAD or FMN as chromophore and is involved in light and temperature sensing in the dark. This again distinguishes the fungal temperature-sensing function from phototropin *in planta*, where temperature changes modulate the photocycle.

## Results

### LreA uses predominantly FAD as chromophore

**Figure 1:**
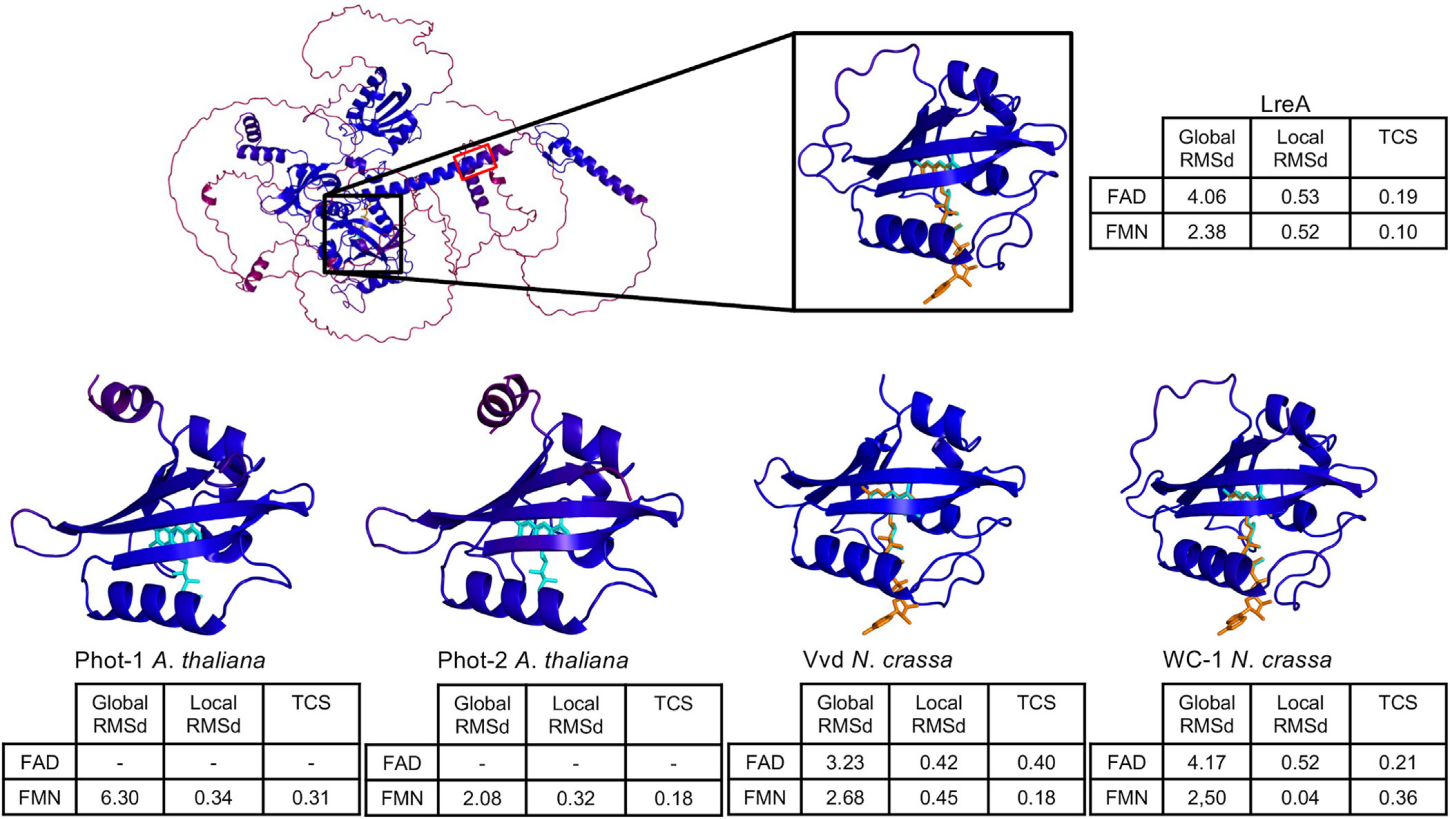
**AlphaFold2and AlphaFill prediction for LreA.** AlphaFold 2 and AlphaFill were used to predict the protein structure and the chromophore. AlphaFold 2 prediction for LreA is shown on *top* with the LOV-domain in more detail on the *right*. For Phot-1, Phot-2, Vvd, and WC-1 only the LOV-domain prediction is shown. Confidence for AlphaFold 2 predictions is based on per-residue scores measured through pLDDT (predicted local distance difference test). pLDDT was also used to color code the residues in PyMOL. The scale on the *bottom* represents pLDDT confidence predictions ranging from 0 to 1, with higher scores corresponding to higher confidence. Confidence for transplant of the chromophore by AlphaFill is represented by Global RMSd (root-mean-square deviation), Local RMSd, and TCS (transparent clash score), with lower values representing higher confidence. FAD is colored *orange*, FMN *turquoise*. The location of the KKKRKRRK sequence in *A. alternata* LreA is indicated by a *red box*. Protein sequences were obtained from UniProt. UniProt ID for LreA: A0A4D6ELU6, Phot-1: O48963, Phot-2: P93025, Vvd: Q9C3Y6, WC-1: Q01371.

To obtain first insights into the structure and the chromophore of LreA we used AlphaFold 2 to model LreA and compared it to the blue-light receptors Phot-1 and Phot-2 from *A*. *thaliana* and Vvd and WC-1 from *N*. *crassa*. The LOV-domain as the core structure is predicted with a high confidence (pLDDT >0.9), which consists of five anti-parallel beta-sheets and 4 to 5 alpha-helices, which tightly surround the chromophore in the binding pocket (Fig. 1).

Next, we extended the model by using AlphaFill to add FAD (turquoise) and FMN (orange) as possible chromophores. While the general structure for all LOV domains was similar, the prediction for the chromophore differed. Both phototropins from *A*. *thaliana* were predicted to be only associated with FMN, while the fungal blue-light receptors were predicted to use either FMN or FAD with higher confidence for FMN (lower global RMSd, local RMSd and TCS) (Fig. 1). The prediction for both phototropins is in agreement with the experimental findings with FMN as chromophore (34, 35). This seems to be different for the fungal receptors. *N. crassa* Vvd appears to use both FMN or FAD, while WC-1 uses FAD as a chromophore (14, 23, 24).

The analysis of the LreA protein sequence also revealed a conserved DBD sequence motif (KKKRKRRK), essential for regulating the circadian clock in *N*. *crassa*, by enabling *frq* transcription and the interaction of WC-1 with FRQ/FRH (36, 37). This sequence motif is located in an exposed C-terminal alpha helix in LreA and is predicted with high confidence (pLDDT >0.9). Although a circadian clock has not yet been documented in *A. alternata*, this conserved motif could be an indication of a central role of LreA in the control of a circadian rhythm (Fig. 1).

**Figure 2:**
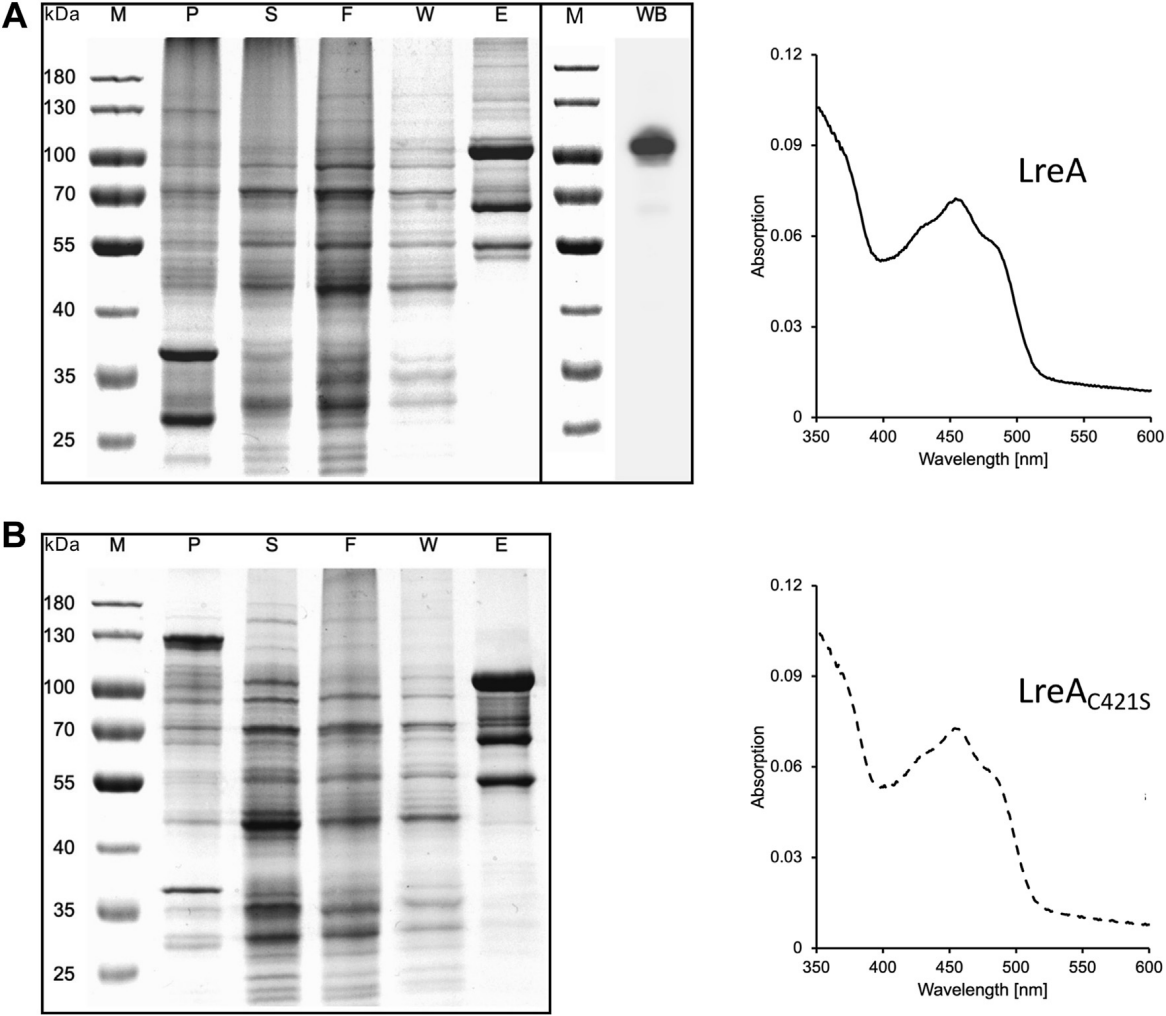
**Heterologous expression of LreA and LreA**_**C421S**_**.***A*, SDS-Page (*left box*) and Western blot of the eluate fraction E (*right box*) to demonstrate heterologous expression and enrichment of LreA. An absorption spectrum of purified LreA (eluate) is shown on the *right*. For heterologous expression of LreA plasmid pHS1 was used. *B*, SDS-Page to demonstrate heterologous expression of LreA_C421S_. An absorption spectrum of LreA_C421S_ is shown on the *right*. For heterologous expression of LreA_C421S_ plasmid pHS2 was used. For the SDS-Page 5 μl of protein marker, 3 μl of pellet- (P), supernatant- (S) and flowthrough- (F) samples, 10 μl of wash- (W) and eluate- (E) samples were loaded. P: Insoluble cell debris after cell disruption, S: Supernatant after centrifugation, F: Flow-through of the supernatant through the column, W: Washing of the column, E: Eluate, WB: Western blot. PageRuler 180 kDa prestained protein ladder from Thermo Fisher Scientific was used as the protein marker (M). The molecular mass of the marker proteins is shown in kDa. LreA and LreA_C421S_ were used at a concentration of 0.7 mg/ml for spectroscopic measurements. The scanning speed was 200 nm/min. Lysis buffer was used for baseline recording. Absorption spectra were recorded under red safety light.

After the modeling approaches, we aimed to study the spectral properties of *A. alternata* LreA. Therefore, we expressed the fungal photoreceptor in *E*. *coli* as a *Strep*-*tag* fusion protein. Although several protein bands were visible after affinity purification, LreA was highly enriched. The apparent molecular mass of LreA was approximately 110 kDa, which is in good agreement with the calculated molecular mass of 114 kDa (38). The absorption spectrum of LreA in its dark-adapted state represented LOV-domain specific features, with an absorption peak at 450 nm and two vibrational bands at 430 and 480 nm, indicating the existence of a flavin chromophore (39, 40) (Fig. 2*A*).

AlphaFold 2 predictions revealed the general structural similarities between blue-light receptors from different kingdoms. Especially the chromophore binding domain showed high structural conservation (Fig. 1). Within the chromophore pocket a cysteine, in close proximity to the chromophore, is highly conserved between all LOV-Domain blue-light receptors, independent of the used chromophore. The cysteine is necessary for signal transduction and forms a covalent bond with the chromophore upon light absorption (40, 41, 42). To further investigate the role of the conserved cysteine beyond light signal transduction, we changed the cysteine (amino acid 421) to serine using site-directed mutagenesis. LreA_C421S_ showed the same flavin-like spectral properties, showing that cysteine 421 is not required for chromophore binding (Fig. 2*B*).

**Figure 3:**
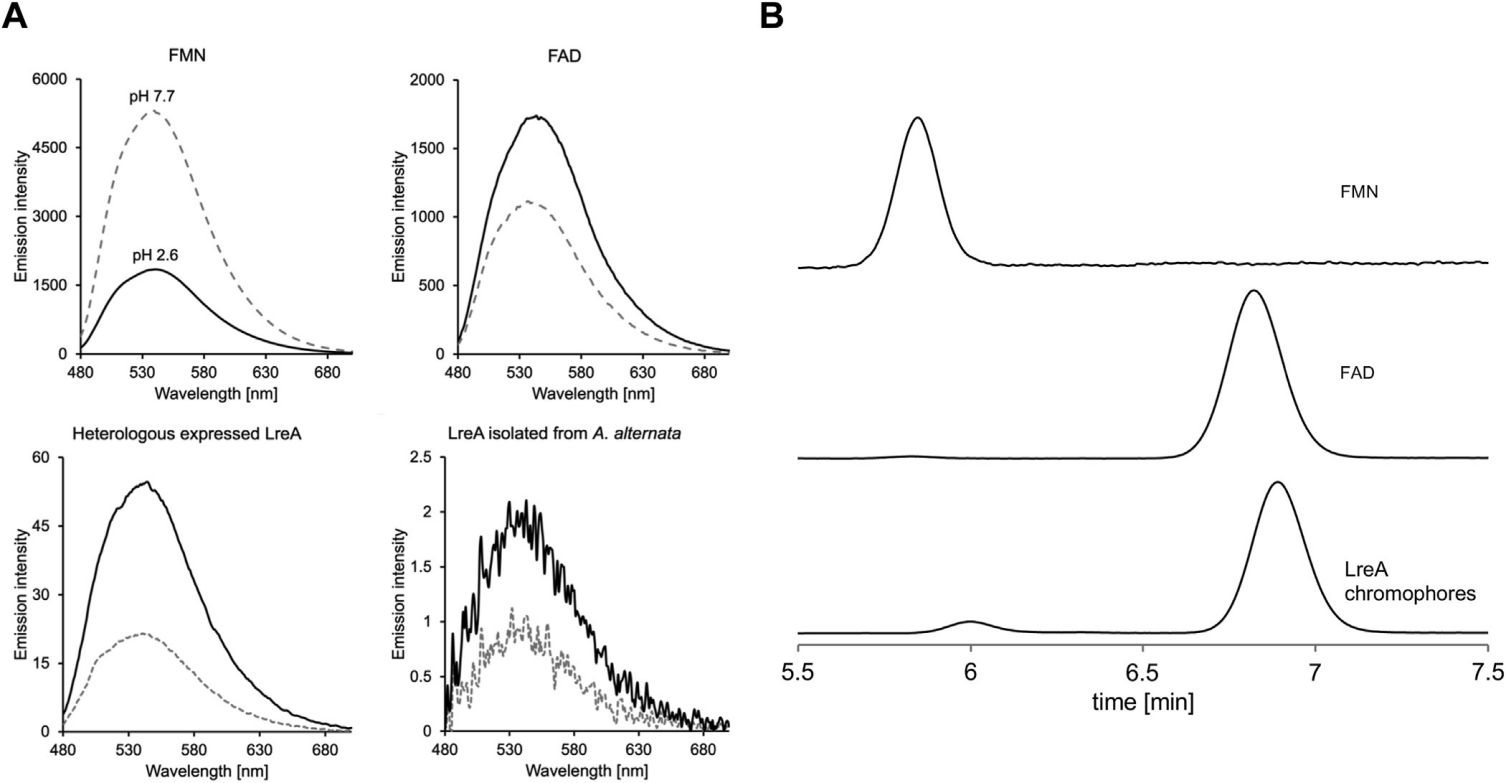
**Determination of the chromophore of LreA.***A*, determination of the chromophore of LreA by fluorescence spectroscopic analysis in comparison to FAD and FMN. The chromophore of LreA was separated from the protein by heating to 95 °C for 3 min and subsequent centrifugation. FMN and FAD were used at a concentration of 10 μM, respectively. All samples were made up of 1 ml of 0.1 M potassium phosphate buffer pH 7.7. The excitation wavelength for emission spectra was 450 nm. Spectra were recorded from 470 to 700 nm. The scanning speed was 200 nm/min, the sensitivity was set to high, excitation and emission bandwidth was 2.5 nm. The samples were acidified with 100 μl of 1 M HCl after the first measurement, and the emission spectrum was recorded again. 0.1 M potassium phosphate buffer pH 7.7 was used for baseline recording. Samples were kept in darkness and measurements were carried out under a red safety light. We repeated all measurements three times. *B*, the determination of the chromophore of LreA by HPLC using A C18 column. We detected FAD, FMN, and the chromophore of LreA at a wavelength of 254 and 450 nm. The chromophore of heterologous expressed LreA was separated from the protein by heating to 95 °C for 3 min and subsequent centrifugation. FAD and FMN were used at a concentration of 100 μM respectively and were heated to 95 °C as well. All samples were made up of water. FAD was used as the flavine-adenine-dinucleotide disodium salt. FMN was used as riboflavin 5′-monophosphate sodium salt hydrate.

To determine whether LreA uses FAD or FMN, we isolated the chromophore(s) from the photoprotein and spectroscopically compared it to FMN and FAD (43). The two flavin derivatives can be easily distinguished after pH shift by fluoresence spectroscopy. The differences in the emission intensity, caused by the pH, are due to different quantum yields in the pH range of 3.0 to 10.0. The additional adenine group of FAD as compared to FMN leads to the formation of an intramolecular complex with the riboflavin molecule at neutral pH. Electron transfer between adenine and flavin within the complex results in decreased quantum yield. A reduction in pH leads to protonation and to a dissolution of the nonfluorescent conformation for FAD and thus to an increase in emission intensity. For FMN, protonation leads to a transition of the neutral flavin into the cationic form, which fluoresces less (44, 45, 46). The isolated chromophore of LreA displayed the same spectral changes as FAD after acidification, suggesting FAD as the chromophore in the fungal protein. The result obtained with the LreA protein isolated from *E. coli* was confirmed with LreA enriched from *A. alternata*. LreA was tagged with an *HA*-*tag* and isolated from the fungal mycelia. We performed the same fluorescence spectroscopic analysis confirming FAD as the chromophore for LreA in the native organism (Fig. 3*A*) As a difference to previous experiments (43) we did not observe a fourfold increase for the emission of FAD after reduction of the pH but only 1.5 fold. This could be due to different working concentrations or other experimental details. Higher FAD concentration lead to a reduced increase in fluorescence at low pH. For FAD isolated from LreA expressed in *E. coli* the factor was close to 3. For LreA isolated from *A*. *alternata*, the concentration was very low and the spectra very noisy. Still the increase of the fluorescence after pH shift was very clear.

Besides the spectroscopic identification, the chromophore was also analyzed by HPLC. We exchanged the buffer with water and isolated the chromophore from the photoprotein as described (43). Upon separation with a C18 HPLC column, the chromatogram showed two distinct peaks with a retention time of 5.998 and 6.890 min. The observed peaks showed similar retention times and spectra as FMN (5.842 min) and FAD (6.797 min) standards, indicating that LreA uses FAD or FMN as chromophores. The small differences in the retention times are likely caused by the detergent IGEPAL CA-630, which was used for protein purification and could not completely be removed by buffer exchange from the LreA sample. This could influence the binding to the column matrix. While both flavins could be detected, the ratio between FAD and FMN was roughly 16 to 1. FAD appears to be the main chromophore of LreA, also explaining why we only detected FAD spectroscopically. This further shows that fungal blue-light receptors show a more diverse spectrum of chromophores, however it is still unknown why different chromophores are used and if they could serve different functions (Fig. 3*B*).

### LreA produces reactive oxygen species and shows self-oxidative damage

**Figure 4:**
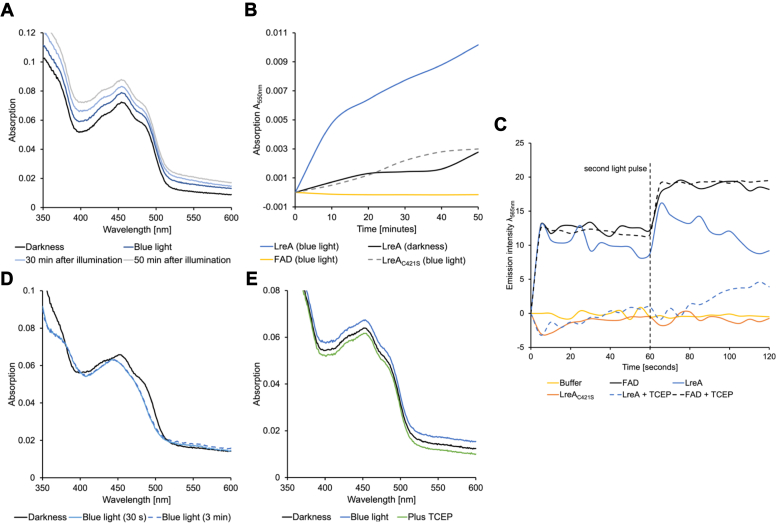
**LreA produces ROS and shows self-oxidative damage.***A*, absorption spectrum of LreA over a 50-min period after blue light illumination. The absorption spectrum was first recorded in darkness. Afterward, the sample was illuminated with blue light for 30 s and an absorption spectrum was recorded immediately after. Further absorption spectra were recorded 30 and 50 min after illumination. *B*, absorption rates at 550 nm were recorded for LreA, LreA_C421S_, and FAD with and without 30 s of blue light illumination over a 50-min period. Absorption was recorded every 10 min. *C*, detection of ROS production by fluorescence spectroscopy for LreA, LreA_C421S_, CryA, and FAD. Samples were supplemented with 1 μl 2.5 mM CellROX Orange Reagent. Samples were first measured in the dark and afterward illuminated with blue light for 30 s. After 60 s of measurement, the samples were again illuminated for 30 s 5 mM of TCEP was used for the respective measurements. Data observed in the dark were applied against values after illumination. Lysis buffer was used for calibration. *D*, the absorption spectrum of LreA with TCEP added before blue light illumination. LreA was first supplemented with 5 mM TCEP and an absorption spectrum was recorded in darkness. Afterward, the sample was illuminated with blue light for 30 s and the absorption was measured. The sample was then illuminated for 3 min. *E*, the absorption spectrum of LreA with TCEP added after blue light illumination. The absorption spectrum of LreA was first recorded in darkness without TCEP. The sample was subsequently illuminated with blue light for 30 s and an absorption spectrum was recorded. Afterward, 5 mM TCEP was added to the sample and a new absorption spectrum was recorded. LreA and LreA_C421S_ were used at a concentration of 0.7 mg/ml respectively. FAD was used at a concentration of 10 μM. All samples were made up of lysis buffer. FAD was used as the flavine-adenine-dinucleotide disodium salt. The scanning speed was 200 nm/min. A lysis buffer was used for baseline recording. Samples were handled under a red safety light to record spectra in darkness. We repeated all measurements three times.

We expressed LreA in *E*. *coli* and examined the isolated protein spectroscopically. Blue light illumination for 30 s led to light scattering in the sample, visible as a shift in the absorption of LreA. Light scattering increased further over 50 min and increased very little if the protein was kept in the dark (Fig. 4, *A* and *B*). We anticipated that photoactivation of LreA with blue light leads to the production of reactive oxygen species (ROS), which in turn causes agglomeration of the protein. To test this hypothesis, we supplemented LreA and FAD with CellROX Orange Reagent to monitor ROS production spectroscopically. CellROX is non-fluorescent in a reduced state but emits orange light at 565 nm upon oxidation by ROS. Pure FAD as well as LreA produced ROS after 30 s of blue light illumination, and a second light pulse after 60 s further increased the ROS production for LreA and FAD (Fig. 4*C*). No ROS production was observed for the cysteine-mutated LreA variant. Likewise, LreA_C421S_ did not agglomerate upon blue-light illumination (Fig. 4, *B* and *C*).

To further strengthen the evidence that ROS causes the agglomeration, we added the reducing agent TCEP. This treatment reduced the production of ROS, and only a second light pulse led to a slight ROS production 20 s after illumination. In comparison pure FAD supplemented with TCEP did not show any difference in ROS production, suggesting that TCEP possibly causes a conformational change in LreA, thereby reducing the access of free O_2_ to the chromophore (Fig. 4*C*). Protein aggregation was not observed even after increased blue-light illumination for 3 min (Fig. 4*D*). The addition of TCEP after blue light illumination reduced the concentration of protein agglomerates, suggesting that the oxidative damage could be reversed with the reducing agent (Fig. 4*E*).

To test whether ROS production is a general feature for blue-light receptors, we studied cryptochrome A from *A*. *alternata*, which we heterologously expressed in *E*. *coli*. No ROS production could be observed for CryA, even after the second blue-light illumination (data not shown).

### LreA is a blue-light receptor

**Figure 5:**
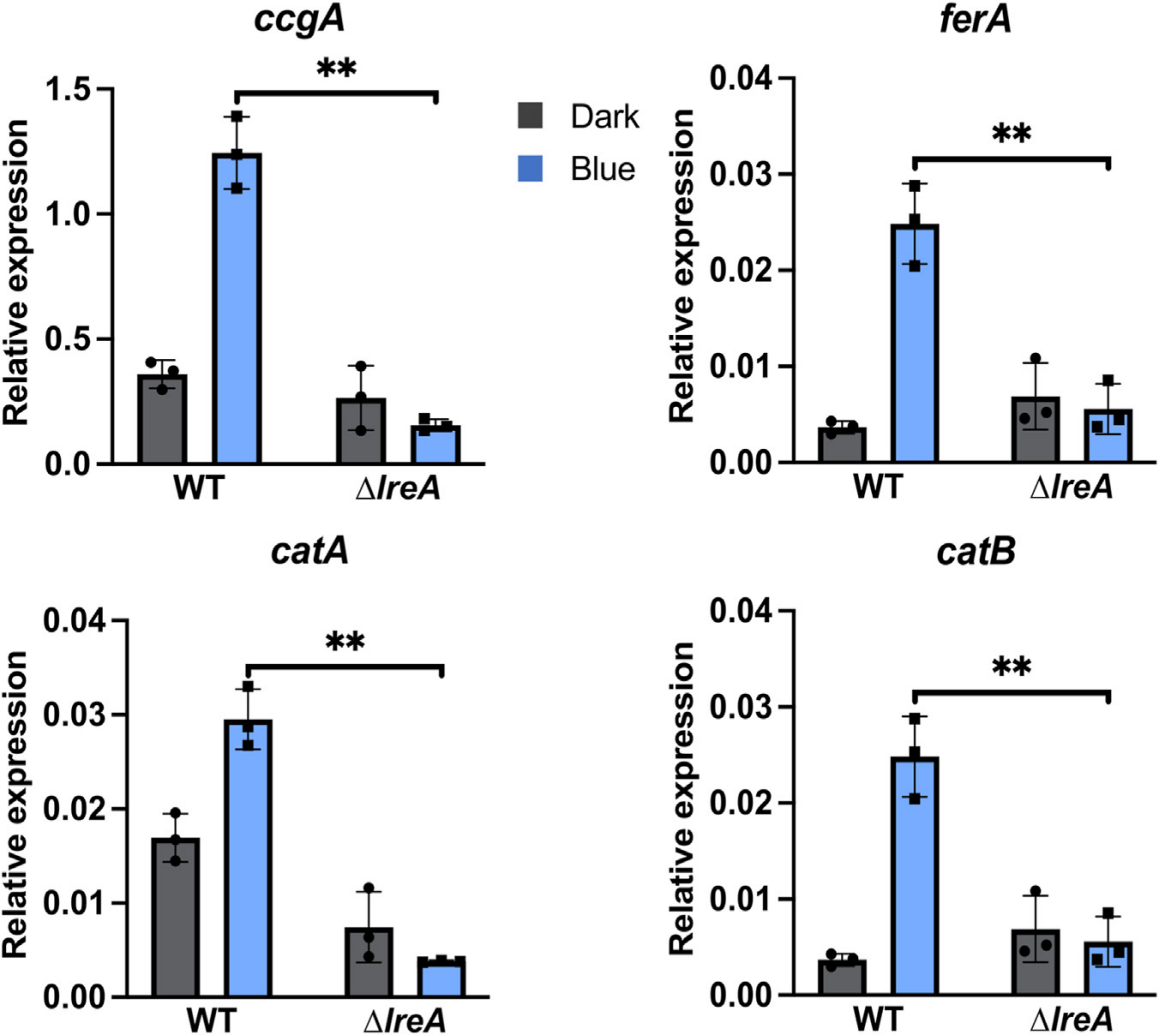
**LreA is a blue light sensor.** Blue-light dependent expression analysis of different light-regulated genes in *A*. *alternata* wild-type strain (WT) and in the *lreA*-deletion strain (sOI3). 50,000 spores of the respective strains were incubated in 20 ml mCDB medium in Petri dishes for 60 h at 28 °C in the dark. The mycelium was either illuminated for 15 min with blue light at 28 °C or kept in darkness for 15 min at 28 °C. Harvesting of the mycelium was done under a green safety light. For the RT-qPCR, 100 ng RNA and 0.4 μM of primers were used per reaction. The *h2b* gene was used for normalization. Error bars represent the standard deviation of three biological replicates and two technical duplicates. Statistical analysis was performed with Student’s test, ∗∗*p* ≤ 0.01. *Dots* and *squares* represent individual data points for the biological triplicates. Relative expression data for darkness are displayed as *grey bars* and *dots*. Data for blue-light illumination are shown by *blue bars* and *squares*.

We next studied the role of LreA as a blue-light receptor in *A. alternata*. We used the wild type and a *lreA*-deletion strain and performed light dependent expression analyses of several genes, which we had characterized before as blue-light responsive in *A*. *alternata* (27). All tested genes were highly upregulated in the mycelium after illumination with blue light. Blue-light induction was significantly reduced in the *lreA*-deletion strain as compared to the wild type, suggesting that LreA is an active blue light receptor in *A*. *alternata* (Fig. 5).

### LreA is involved in temperature sensing

**Figure 6:**
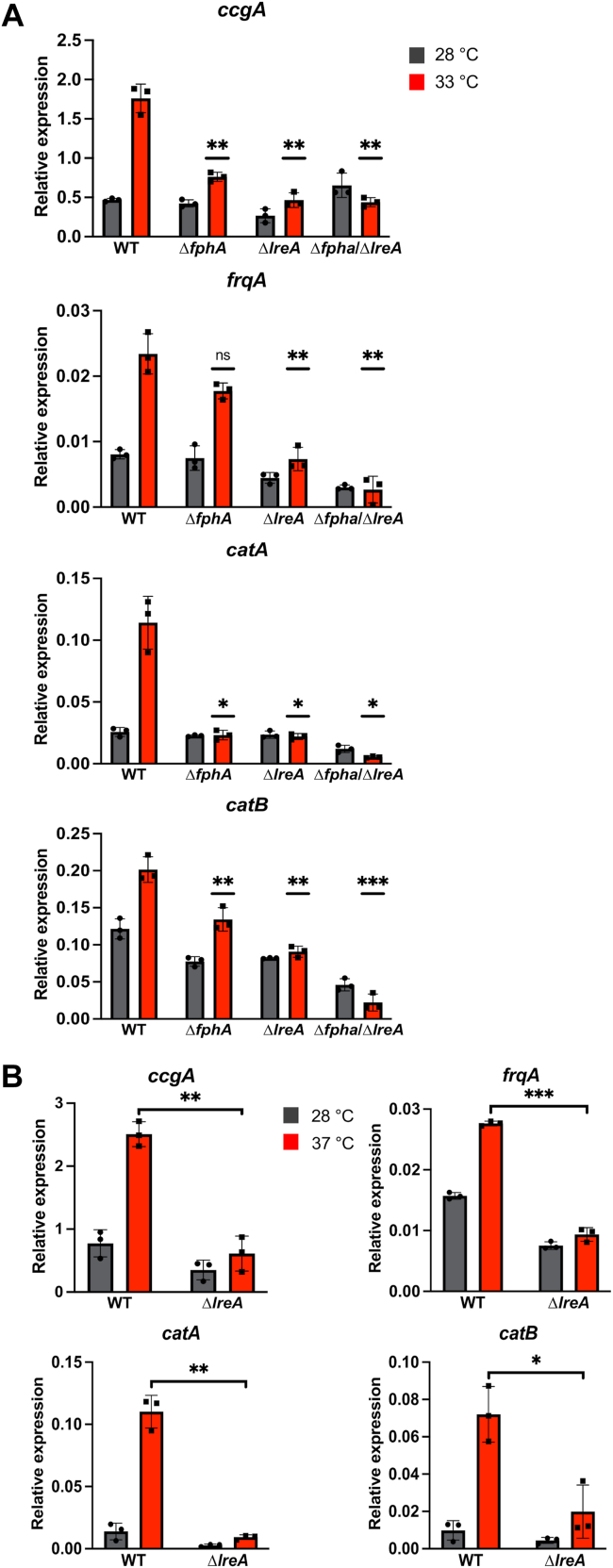
**LreA is involved in temperature sensing.***A*, temperature-dependent expression analysis of different transcription and developmental marker genes at 28 and 33 °C in *A*. *alternata* wild-type strain (WT), in the *lreA*-deletion strain (sOI3), in the *fpha*-deletion strain and the double deletion strain (sJJG01). 50,000 spores of the respective strains were incubated in 20 ml mCDB medium in Petri dishes for 60 h at 28 °C in the dark. The mycelium was transferred onto the surface of prewarmed mCDB medium at 28 and 33 °C and incubated for 15 min in darkness in an incubator at the respective temperature. *B*, temperature-dependent expression analysis of different transcription and developmental marker genes at 28 and 37 °C in *A*. *alternata* wild-type strain (WT) and in the *lreA*-deletion strain (sOI3). 50,000 spores of the respective strains were incubated in 20 ml mCDB medium in Petri dishes for 60 h at 28 °C in the dark. The mycelium was transferred onto the surface of prewarmed mCDB media at 28 and 37 °C and incubated for 15 min in darkness in an incubator at the respective temperatures. Both experiments were performed under a green safety light. For the RT-qPCR, 100 ng RNA and 0.4 μM of oligonucleotides were used per reaction. The *h2b* gene was used for normalization. Error bars represent the standard deviation of three biological replicates and two technical duplicates. Statistical analysis was performed with the student’s test, ∗*p* ≤ 0.05; ∗∗*p* ≤ 0.01; ∗∗∗*p* ≤ 0.001. *Dots* and *squares* represent individual data points for the biological triplicates. Relative expression data for 28 °C is represented by *grey bars* and *dots*. Data for 33 and 37 °C is represented by *red bars* and *squares*.

To test whether LreA is involved in temperature sensing, we used a *lreA*-deletion strain, a phytochrome *fphA-*deletion, and a *lreA/fphA* double-deletion strain and performed temperature-dependent expression analysis of several genes known to respond to ambient temperature changes in *A. alternata* and/or *A. nidulans* (28). All genes were highly upregulated in the mycelium after a temperature shift from 28 to 33 °C in the dark. Heat-stress induction was significantly reduced in the *lreA*-deletion strain as compared to the wild type, suggesting that LreA contributes to temperature sensing in *A*. *alternata*. Similar to the *lreA*-deletion strain, the expression in the *fphA*-deletion strain was greatly reduced, except for the expression of *frqA*. The double-deletion strain showed for all genes a further reduction in the temperature-induced expression, for *catA* almost a complete abolishment (Fig. 6*A*).

In order to test the robustness of the temperature-sensing function of LreA, we studied also a temperature shift from 28 to 37 °C. The expression for all genes was still highly upregulated, with similar expression levels for *frqA* and *catA*, an increased expression for *ccgA*, and a decrease in the expression for *catB*. The *lreA*-deletion strain still showed the same reduced expression levels, indicating a broad spectrum for the temperature sensing capabilities of LreA (Fig. 6*B*).

### Cysteine 421 is necessary for temperature sensing

**Figure 7:**
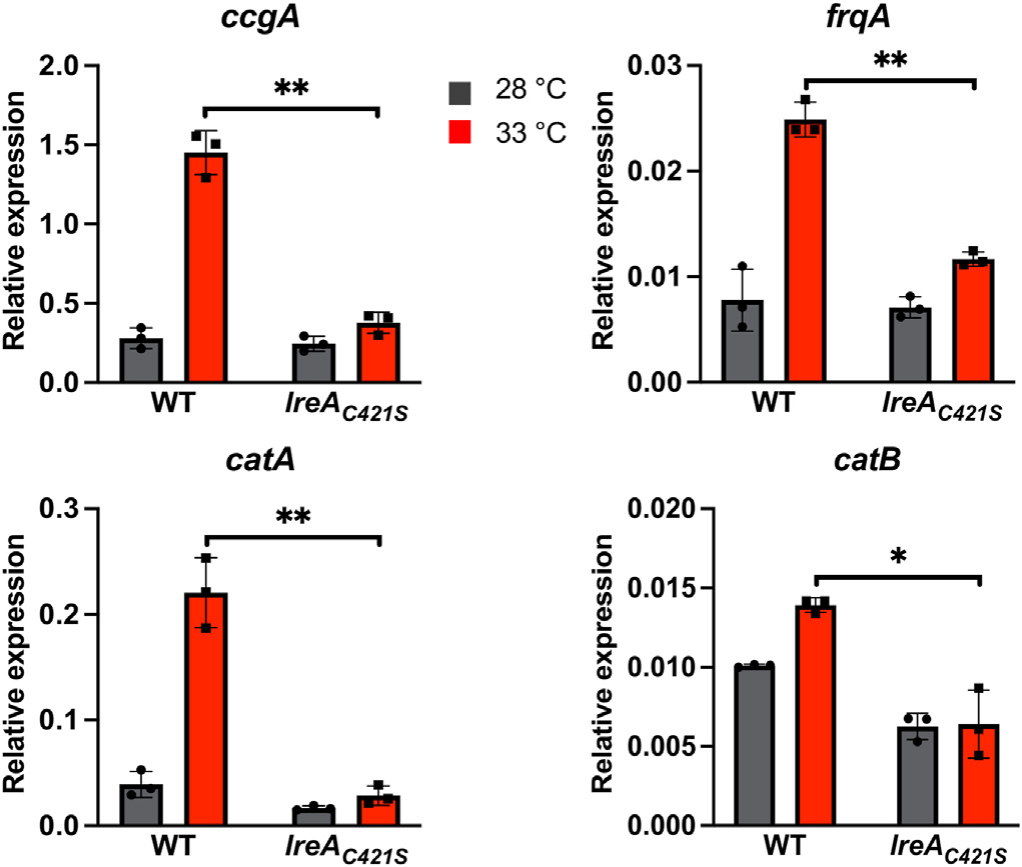
**Cysteine 421 is necessary for temperature sensing.** Expression analysis of different transcription and developmental marker genes at 28 and 33 °C in *A*. *alternata* wild-type strain (WT) and in the *lreA*_*C421S*_ strain (sHS1). 50,000 spores of the respective strains were incubated in 20 ml mCDB medium in Petri dishes for 60 h at 28 °C in the dark. The mycelium was transferred onto the surface of prewarmed mCDB medium at 28 and 33 °C and incubated for 15 min in the dark at the respective temperatures. Experiments were done under a green safety light. For the RT-qPCR, 100 ng RNA and 0.4 μM of primers were used per reaction. The *h2b* gene was used for normalization. Error bars represent the standard deviation of three biological replicates and two technical duplicates. Statistical analysis was performed with Student’s test, ∗*p* ≤ 0.05; ∗∗*p* ≤ 0.01. *Dots* and *squares* represent individual data points for the biological triplicates. Relative expression data for 28 °C is represented by *grey bars* and *dots*. Data for 33 °C is represented by *red bars* and *squares*.

We previously reported on the possible mechanism of FphA temperature sensing in *A. nidulans*, indicating that temperature increase goes along with conformational changes and a softening of the chromophore binding pocket, possibly mimicking photoactivation (28). Blue-light receptors like LreA have a conserved cysteine in close proximity to the chromophore that is essential for light sensing (40, 41, 42). The cysteine 421 was not necessary for the attachment of the chromophore in LreA but played a major role in the production of reactive oxygen species, which goes along with light sensing. We found that the mutated *lreA*_*C421S*_ strain phenocopied the *lreA*-deletion strain, indicating an essential role for the chromophore or the conserved cysteine for temperature sensing (Fig. 7).

### Temperature sensing of LreA is not necessary for activation of the heat-shock response

**Figure 8:**
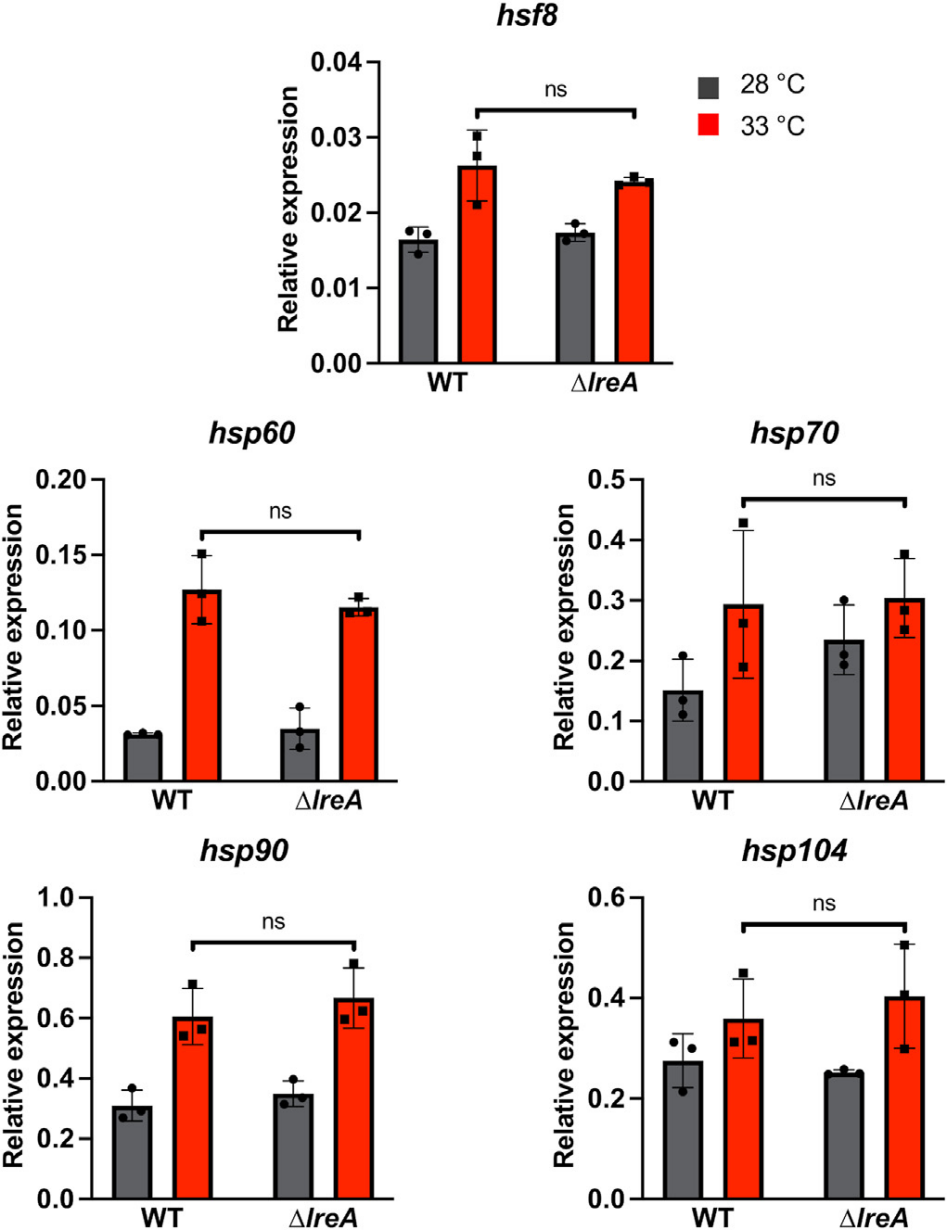
**Temperature sensing of LreA is not necessary for activation of the heat-shock response.** Expression analysis of the regulator *heat*-*shock factor 8* (*hsf8*) and different heat-shock proteins (HSPs) at 28 and 33 °C in *A*. *alternata* wild-type strain (WT) and *lreA*-deletion strain (sOI3). 50,000 spores of the respective strains were incubated in 20 ml mCDB medium in petri dishes for 60 h at 28 °C in the dark. The mycelium was transferred onto the surface of prewarmed mCDB medium at 28 and 33 °C and incubated for 15 min in the dark at the respective temperatures. The execution took place under a green safety light. For the RT-qPCR, 100 ng RNA and 0.4 μM of primers were used per reaction. The *h2b* gene was used for normalization. Error bars represent the standard deviation of three biological replicates and two technical duplicates. Statistical analysis was performed with Student’s test, ns, *p* > 0.05. *Dots* and *squares* represent individual data points for the biological triplicates. Relative expression data for 28 °C is represented by *grey bars* and *dots*. Data for 33 °C is represented by *red bars* and *squares*.

A sudden temperature increase leads to the so-called heat-shock response, a conserved stress response among organisms. This mechanism is triggered by unfolded or misfolded proteins. The consequences of an increase in temperature result in the activation of one or more heat-shock factors (HSF) and the expression of heat-shock proteins (HSP) (47, 48, 49). We tested if the temperature sensing of LreA also influences the heat-shock response in *A*. *alternata* and found that *hsf8*, *hsp60*, *hsp90* were highly upregulated in the mycelium of *A. alternata* after a temperature shift from 28 to 33 °C in the dark, while *hsp70* and *hsp104* showed a minor increase in the expression level. The *lreA*-deletion strain didn’t show any significant differences in the expression levels compared to the wild type, suggesting that the conserved heat-shock response and the temperature sensing by LreA are two different temperature-sensing mechanisms (Fig. 8).

## Discussion

Light and temperature are two important environmental cues for most living organisms. Light not only serves as an energy source for photosynthetic organisms but is also used for orientation, communication and adaptation. Likewise, fungi use light as an indicator for the growth of their mycelium at air-exposed surfaces, which triggers for instance fruiting body or spore formation (10, 50). Such a function could be fulfilled with just one wavelength and one corresponding photoreceptor. Nevertheless, many fungi use photoreceptors for blue-, green-, and red light (10). This suggests rather complex light perception abilities and different biological responses. Fungi are likely not only able to detect ambient light but possibly also change light compositions from dusk to dawn. Along with the light-spectral composition typically the temperature changes. There is evidence that plant and fungal phytochrome and plant phototropin are able to respond to ambient temperature changes (28, 29, 30). Here, we describe the dual-function LOV-domain protein LreA of *A*. *alternata* as light- and temperature sensor. As a difference to plant phototropin the temperature-sensing function is light-independent. This leads us to speculate that the temperature-sensing function may be the ancient function of the flavin-containing photoreceptor.

The ability of FAD and FMN to produce ROS has been previously described. Illumination with blue light leads to the formation of the excited triplet state with about 200 kJ mol^−1^ energy. The stored energy can be transferred to O_2_ by the dexter mechanism, creating oxidant singlet oxygen (51, 52). We confirmed the production of ROS for pure FAD and also FMN (data not shown) and activation of LreA by blue light led to the same immediate production of ROS, thereby causing self-oxidative damage and agglomeration of the protein. Such a connection between illumination and ROS production has only been reported for some blue-light receptors, and thus appears not to be a general phenomenon. Likewise, cryptochrome A from *A*. *alternata* failed to produce any ROS. In most cases, ROS formation for blue light receptors has only been described with a mutated cysteine, which normally should quench the triplet state and inhibit ROS formation. However, ROS formation could be detected for *A. alternata* LreA without any mutation. Similar to *A. alternata* LreA, the fungal blue-light receptor Vivid from *N*. *crassa* and several plant cryptochromes are able to produce ROS without any mutation (32, 53). While it is assumed that ROS produced by cryptochromes is used as a signal molecule to regulate gene expression, we rather propose that ROS production by LreA regulates the activity and protein availability *in vivo*, similar to Vvd from *N*. *crassa* (31, 32, 53, 54). *A. alternata* LreA is not known to be regulated by another blue-light receptor-like Vivid (Vivid is absent), this could explain why a self-regulated mechanism is necessary to adjust the activity of LreA. In line with this explanation, the reducing agent TCEP prevented ROS production for a short time, possibly by causing a conformational change in LreA reducing the access of free O_2_ to the chromophore or by causing a different energy transfer within the protein. In comparison, the amount of ROS detected for free FAD was not affected by TCEP. An effect of TCEP directly on the chromophore or on ROS is therefore unlikely. While the ROS production was prevented at first, we found that a second light pulse after 60 s led again to the production of ROS, although it was still reduced in comparison to the untreated sample. This could indicate that ROS production over time always occurs to prevent permanent activation of LreA. We assume that the quenching of the triplet state is therefore not always effectively happening leading to the formation of ROS over time. We could show that besides reducing the ROS production, adding TCEP after blue light illumination also reversed the agglomeration. Agents like TCEP are able to reduce disulfide bonds. ROS therefore probably leads to the formation of such bonds, causing agglomeration, which inhibits further activation of the protein after a certain time. This self-regulating mechanism seems to be dependent on the conserved cysteine 421 because no ROS production was observed for the cysteine-mutated LreA variant. Likewise, LreA_C421S_ did not agglomerate upon blue-light illumination. This distinguishes *A. alternata* LreA from Vivid from *N*. *crassa*, which was still able to produce ROS despite the mutated cysteine (32). It has been previously described that the active and dark-adapted states of blue-light receptors can have different accessibility of oxygen to the chromophore. Targeted mutations can also influence this accessibility (51, 55, 56). We therefore hypothesize that the mutation causes a permanent conformational change totally inhibiting the access of free O_2_ to the chromophore, in comparison to a short-term conformational change by the reducing agent TCEP. Although we assume that ROS production is mainly required for the regulation of LreA activity, it cannot be ruled out that similar to some cryptochromes, ROS is also used for signal transduction.

Temperature is an environmental factor that is subject to constant fluctuations and can affect the survival of an organism by affecting DNA, enzyme- or membrane stability. Therefore, the perception of temperature and the corresponding reaction to temperature changes is an essential property of all organisms (57, 58). In recent years, photoreceptors have been discovered as an entirely new class of temperature sensors. In plants, bacteria, and fungi, it could be shown that some red- and blue-light receptors can also function as temperature sensors in addition to their light-sensing function. While the mechanism of temperature perception by plant and bacterial photoreceptors is based on temperature-dependent altered lifetimes of the photoactive states, a light-independent temperature perception has been described for the *A. nidulans* and *A. alternata* phytochromes (28, 29, 33, 59, 60). In this study, we found that LreA also acts as a light and temperature sensor. The temperature-induced expression of catalase genes, the clock-controlled gene A, and the frequency gene *frqA* were reduced in the absence of LreA. Frequency A plays a major role in the regulation of the circadian clock in *N*. *crassa*. The clock can be entrained by light and by temperature (61, 62, 63). Hence, LreA could be the crucial protein for both environmental cues. The presence of the conserved DBD sequence motif (KKKRKRRK) in LreA, which is necessary for the regulation of circadian rhythms by WC-1 in *N*. *crassa*, further provides evidence for an essential role for LreA in a putative circadian clock in *A*. *alternata*. Although a circadian clock has not yet been clearly demonstrated in *A. alternata*, the genome of *A*. *alternata* harbors the genes known to be involved in the circadian rhythm in *N*. *crassa*. While the *fphA*-deletion strain showed similar results to the *lreA*-deletion, we found that it did not influence the expression of *frqA*, indicating a minor role for FphA in a possible circadian clock control in *A*. *alternata*. Nonetheless, we propose that both LreA and FphA function as temperature sensors in *A*. *alternata*. We previously already showed an interaction between LreA and FphA in light regulation (through LreB) (26). Taken together, our data suggest a possible interplay between FphA and LreA, not only in light perception but also in temperature perception in *A*. *alternata*.

While the mechanism for light-dependent temperature perception has been studied well in plants and bacteria, the mechanism for light-independent temperature perception is still largely unknown and has only been studied in a few organisms. We already proposed that the photoreceptors FphA and LreA harbor functions independent of light perception in *A*. *alternata*, a light-independent and temperature-dependent activity for the protein therefore seems plausible (27). We previously reported on the possible mechanism of FphA temperature sensing in *A. nidulans*, indicating that heat stress could mimic photoactivation (28). We tested this hypothesis for LreA by altering the conserved cysteine, which is essential for light-sensing. The mutated *lreA*_*C421S*_ strain phenocopied the *lreA*-deletion strain, indicating an essential role for the chromophore or the conserved cysteine for temperature sensing. In this work we showed that blue-light dependent ROS production of LreA depends on the conserved cysteine and hypothesize that the mutation causes a conformational change, which renders the protein non-functional for temperature sensing. Therefore, temperature sensing could be independent of the chromophore but depends on conformational changes to activate the protein, thereby mimicking photoactivation.

We hypothesize that LreA acts as a temperature sensor. However, given that LreA acts also as a transcriptional activator, it could be that an unknown temperature sensor upstream of LreA mediated the responses in our experiments. Likewise, if mutation of the critical cysteine 421 causes conformational changes, these could also affect DNA binding of the protein or interaction of LreA at the promoter with other factors or the RNA polymerase. Nevertheless, even in this case, LreA can be considered as a temperature sensor because the response to the temperature would at least contribute to the temperature sensing process.

Our data point to a significantly more complex process for temperature perception as compared to light perception. At least two photoreceptors, LreA and FphA, are involved in temperature sensing in *A*. *alternata*. Furthermore, it was shown that the heat shock response is not affected by the temperature perception of LreA in *A*. *alternata*. This indicates that several different processes and mechanisms for temperature perception have evolved, similar to different light receptors and it seems unlikely that there is one global temperature regulator controlling all temperature-dependent processes. It will be a challenge for future research to decipher the contribution of each sensing system to temperature sensing and their molecular interplay.

## Experimental procedures

### Strains, culture conditions, plasmids and oligonucleotides

*A. alternata* cultures were grown on modified Czapek Doth broth (mCDB) agar (4% glucose, 0.1% yeast extract, 0.1% NaNO_3_, 0.025% NH_4_Cl, 0.1% KH_2_PO_4_, 0.025% KCl, 0.025% NaCl, 0.05% MgSO_4_ 7× H_2_O, 0.001% FeSO_4_ 7× H_2_O, 0.001% ZnSO_4_, and 1.5% agar-agar) and incubated for 10 days at 28 °C. Liquid cultures were also incubated in mCDB at 28 °C, but incubation times varied depending on the application: either 17 h shaking (transformation of *A*. *alternata*) or 60 h without shaking to produce hyphal mats (isolation of RNA). Strains are listed in Table S1 (Supporting Information).

Plasmids were cloned using the NEB NEBuilder HiFi DNA Assembly Cloning Kit. Plasmids are listed in Table S2 (Supporting Information) and oligonucleotides in Table S3 (Supporting Information).

*E*. *coli* strain BL21 DE3 was used for protein expression and top ten cells were used for plasmid replication. Solid or liquid lysogeny broth (LB) medium (tryptone 10 g/l, yeast extract 5 g/l, NaCl 10 g/l, agar-agar 15 g/l) was used for the cultivation of bacterial cultures. For protein expression, LB media was additionally supplemented with 2.5 mM betaine and 100 mM sorbitol. Liquid cultures were incubated in a shaking incubator at 37 °C and 180 rpm. The incubation time for liquid cultures with Top ten cells was 17 h. For BL21 DE3 cells, the incubation time was dependent on the cell density. Bacteria on LB agar were incubated at 37 °C for 17 h. Strains are listed in Table S1 (Supporting Information).

### CRISPR/Cas9 plasmid constructions for deletion of lreA

For the deletion of *lreA* two protospacer sequences were cloned in the pFC332 vector, to produce two different sgRNAs. The design of the construct was done as described (64). Cloning of the plasmids was done in a NEBuilder reaction. *E. coli* transformation and plasmid isolation were done according to standard protocols. Transformation of *A*. *alternata* was achieved by protoplasting of the mycelia as described (64). Positive transformants were confirmed by PCR.

### Complementation of LreA

For complementation of the *lreA*-deletion strain sOI3 with the *lreA* wild type gene, the *lreA* gene was amplified containing 1.2 kb downstream of the open reading frame by PCR using genomic DNA of *A*. *alternata* as template. The PCR fragment was cloned into the linearized plasmid pLS32 containing *A*. *alternata gpdA* as a promoter, an N-terminal 3× *HA*-*tag* for the *lreA* gene, and an ampicillin resistance cassette. The plasmid was co-transformed with pHS14 (*hph*; *Amp*^*R*^) in sOI3 by protoplast transformation (sLS2). Site-directed mutagenesis for *lreA*_*C421S*_ was achieved by PCR. The PCR fragment contained 1 kb up- and downstream of the *lreA*-wild type gene and was cloned into the linearized pJET1.2. The plasmid was co-transformed with pHS14 (*hph*; *Amp*^*R*^) in sOI3 by protoplast transformation (sHS1).

Cloning of the plasmids was done in a NEBuilder reaction. *E. coli* transformation and plasmid isolation were done according to standard protocols. Transformation of *A*. *alternata* was achieved by protoplasting of the mycelia (64). Positive transformants were confirmed by PCR.

### RNA isolation from *A. alternata* and reverse transcription quantitative PCR (RT-qPCR)

Twenty milliliter liquid mCDB medium in a Petri dish was inoculated with 50,000 spores harvested from a 10-day-old mCDB agar plate. Incubation took place in the dark for 60 h at 28 °C. Subsequently, the mycelium was harvested under a green safety light. For temperature-dependent expression analysis, the mycelium was transferred to a Petri dish with prewarmed mCDB medium at 28, 33 or 37 °C and incubated for 15 min in darkness in an incubator at the respective temperature. For blue-light dependent expression analysis, the mycelium was incubated for 15 min under blue light at 28 °C. The mycelium was then dried with Miracloth and paper towels and transferred to a 2 ml reaction vessel. The reaction vessel was then frozen in liquid nitrogen. The amount of harvested mycelium was determined in the reaction vessel by weighing. The mycelium was ground twice in a precooled mortar and then transferred to a 15 ml centrifuge tube with a remainder of liquid nitrogen. After the liquid nitrogen had evaporated, 1 ml of Trizol was added per 100 mg of mycelium and the centrifuge tube was vortexed to resuspend the mycelium. 15 mg of the mycelium were transferred to a new 2 ml reaction vessel. The volume was made up to 1 ml with Trizol. The samples were incubated for 5 min at room temperature. Then 200 μl of chloroform was added, and the samples were vortexed and incubated for 3 min at room temperature. The samples were centrifuged for 15 min at 13,000 rpm at 4 °C. The mixture was separated into three different phases by centrifugation. Four hundred microliter of the upper aqueous phase, which contained the RNA, were transferred to a new 1.5 ml reaction vessel. The RNA was precipitated by adding 500 μl of isopropanol. The samples were incubated at 4 °C for 30 min. Centrifugation was carried out at 13,000 rpm at 4 °C. The supernatant was discarded. The precipitated RNA was washed with 1 ml 70% ethanol. The samples were centrifuged for 5 min at 13,000 rpm and 4 °C. The supernatant was removed and discarded. The RNA was dried for 20 min at room temperature to allow residual ethanol to evaporate. The RNA was dissolved in 50 μl of nuclease-free water. RNA was treated with a TURBO DNA-free kit and diluted to 50 ng μl^−1^. The Luna Universal One-Step RT-qPCR Kit (NEB) was used for the qRT-PCR according to the manufacturer's instructions. Per reaction 100 ng of RNA and 0.4 μM oligonucleotides were used. The program started with 10 min reverse transcription reaction at 55 °C, followed by 1 min at 95 °C for denaturation and 40 cycles of polymerase chain reaction (10 s at 95 °C and then 30 s at 59 °C). Melting curve analysis was carried out for 40 cycles (95–55 °C with 5 s per step). The *h2b* gene was used for normalization. Each transcript level is the average of three biological and two technical replicates.

### Protein extraction of LreA from *A. alternata*

To isolate LreA from *A*. *alternata* we complemented the *lreA*-deletion strain sOI3 with the *lreA* gene, containing an N-terminal 3× *HA*-*tag*. 300 ml of mCDB medium were inoculated with spores of a 10-day-old agar plate. The culture was incubated for 17 h at 28 °C and 180 rpm. On the next day 50 μl of G-agarose beads were centrifuged at 5000 rpm, 4 °C for 30 s in a 1.5 ml reaction vessel. The supernatant was removed and discarded. The G-agarose beads were then washed three times with protein extraction buffer (150 mM NaCl, 100 mM Tris-HCl, 0.05% Tween20) supplemented with 1 mM phenylmethylsulfonyl fluoride (PMSF). The supernatant was discarded each time. 100 μl of protein extraction buffer was added to the G-agarose beads. To harvest the mycelium, 1 ml protein extraction buffer containing 1 mM PMSF was initially placed in a 2 ml reaction vessel. The mycelium was filtered through Miracloth, dried with paper towels, and then ground in a precooled mortar with liquid nitrogen. The ground mycelium was transferred to the 2 ml reaction vessel and inverted. The mixture was incubated on ice for 20 min and inverted every 5 min. The mixture was then centrifuged at 13,000 rpm and 4 °C for 15 min. The supernatant was removed was mixed with 2 μg of anti-HA antibody. The mixture was rotary incubated for 1 h at 4 °C. Then the protein-antibody solution was added to the prepared G-agarose beads. The solution was incubated rotating at 4 °C for 3 h. The probe was then centrifuged for 30 s at 5000 rpm and 4 °C. The supernatant was discarded. The G-agarose beads were again washed three times with 1 ml protein extraction buffer containing 1 mM PMSF. The supernatant was discarded each time. In the end, 1 ml of the designated buffer was added to the G-agarose beads. All steps were performed under a red safety light or in the dark.

### Recombinant expression in *E. coli* and purification of LreA and LreA_C421S_

The *E*. *coli* strain BL21 DE3 was used for heterologous protein expression. We used the pASK-iab3plus vector. pASK contains a *tet* promoter, a C-terminal *strep*-*tag* (*strep*-*tag* II, IBA Lifesciences) for enriching the protein of interest, and an ampicillin resistance cassette. *E. coli* transformation and plasmid isolation were done according to standard protocols. Heterologous expression and purification were identical for LreA and LreA_C421S_. A 50 ml LB preculture supplemented with 50 μg ml^−1^ ampicillin was inoculated with a transformed *E*. *coli* BL21 DE3 colony. The culture grew for 16 h at 37 °C and 180 rpm. On the following day, a 2-l LB main culture, mixed with 50 μg ml^−1^ ampicillin, was inoculated with 1% (v/v) of the pre-culture. The media was additionally supplemented with 2.5 mM betaine and 100 mM sorbitol. The culture was grown at 37 °C and 180 rpm until the optical density at a wavelength of 600 nm reached 0.6. The subsequent addition of 0.2 μg/ml AHT resulted in the induction of the *tet* promoter and thus the protein expression of LreA/LreA_C421S_. Protein expression was carried out at 15 °C and 180 rpm for 20 h. The following day, the culture was centrifuged twice at 9000 rpm and 4 °C for 10 min. The supernatant was discarded, and the pellet resuspended twice in 30 ml lysis buffer (50 mM Tris, 20 mM NaCl, 2% Igepal CA-630) containing 1 mM PMSF and EDTA-free Pierce Protease Inhibitor Tablets. The cells were disrupted twice at 1000 to 1500 bar using the Emulsiflex-C3 high-pressure homogenizer (Avestin Europe GmbH). In order to separate insoluble components, the probe was centrifuged at 18,000 rpm and 4 °C for 1 h. The supernatant was incubated with 40 μg/ml avidin for 20 min. The target protein was then isolated and purified by affinity chromatography using Äktapure and a 5 ml StrepTrap HP column according to the manufacturer’s instructions. For the elution we supplemented the lysis buffer with 5 mM desthiobiotin. Subsequent to the affinity chromatography, the desthiobiotin used for the elution was removed from the eluate using a PD-10 desalting column (GE Healthcare). The procedure was carried out according to the manufacturer's instructions. A vivaspin ultrafiltration unit 30,000 MWCO (molecular weight cut off) (Sartorius) was used to concentrate protein samples. The procedure was carried out according to the manufacturer's instructions. Protein concentrations were determined using the QuBit spectrofluorometer (Invitrogen) according to the manufacturer's instructions. Following the affinity chromatography, a PD-10 desalting column (GE Healthcare) was used for a buffer change. The procedure was carried out according to the manufacturer's instructions. All steps were performed under red safelight or in the dark.

### SDS-PAGE and immunoblot detection

SDS-PAGE was performed according to Laemmli (65). For the SDS-PAGE the Mini-PROTEAN Tetra Vertical Electrophoresis Cell and Power Pac Basic electrophoresis system from Bio-Rad were used. The stacking gel had a concentration of 5% (for two stacking gels: 0.83 ml 30% acrylamide-bisacrylamide, 0.05 ml 10% SDS, 3.4 ml ddH_2_O, 0.63 ml 0.5 M Tris-HCl pH 6.8, 0.05 ml 10% APS, 0.007 ml TEMED), the concentration of the separating gel was 10% (for two separating gels: 3.3 ml 30% acrylamide-bisacrylamide, 0.1 ml 10% SDS, 4 ml ddH_2_O, 0.1 ml 1.5 M Tris-HCl pH 8.8, 0.1 ml 10% APS, 0.01 ml TEMED). The SDS gel was in SDS running buffer (25 mM Tris, 0.1% SDS, 192 mM Glycin) during the electrophoresis. The samples were mixed with 5× sample buffer (200 mM Tris-HCl pH 6.8, 10% SDS, 20% of 40% Glycerol, 0.05% bromophenol blue, 10 mM DTT), incubated for 10 min at 95 °C and centrifuged for 10 min at 13,000 rpm. PageRuler 180 kDa prestained protein ladder from Thermo Fisher Scientific was used as the protein marker. The samples were concentrated in the stacking gel for 15 min at 80 V and separated in the separating gel at 100 V for 2 h. Either a Western blot was performed following the SDS-Page, or the gel was stained for viewing. For staining, the separating gel was stained in 25 ml Coomassie brilliant blue staining solution (45% Methanol, 10% of 100% acetic acid, 45% ddH_2_O, 0.1% Coomassie Brilliant Blue R-250) for 30 min. Destaining was carried out in Coomassie brilliant blue destaining solution (40% methanol, 10% of 100% acetic acid, 50% ddH_2_O). Following the SDS-PAGE, the samples were electrotransferred to a nitrocellulose membrane. The transfer was performed at 80 V and 4 °C for 2 h. The membrane was incubated overnight rotating with PBS blocking buffer (4 mM KH_2_PO_4_, 16 mM Na_2_HPO_4_, 115 mM NaCl) and 1% (w/v) milk powder in a 50 ml centrifuge tube at 4 °C. The following day, the membrane was rotary-washed three times with 2 ml PBS-T (PBS supplemented with 0.1% Tween20 (v/v)) for 5 min. For antibody binding, 4 μl of strepMAB-classic HRP conjugate were incubated with 10 ml of PBS-T buffer. Antibody binding was carried out rotating for 1 h at room temperature. The membrane was then washed three times with 20 ml PBS-T buffer for 5 min at room temperature. The membrane was then washed three times with PBS buffer for 5 min at room temperature. Finally, the Western blot was examined through chemiluminescence. The membrane was incubated with a solution of 1 ml solution A (50 ml 0.1 M Tris-HCl pH 6.8, 12 mg Luminol, 10 ml DMSO), 100 μl solution B (10 ml DMSO, 11 mg p-Hydroxycoumarin acid) and 0.3 μl H_2_O_2_ and examined and photographed on a Chemismart-5000 (Peqlab).

### Spectroscopy

All spectroscopic measurements were carried out in a 700 μl quartz cuvette at room temperature. Absorption spectra were recorded with the JASCO V-750 spectrophotometer (JASCO GmbH). Emission spectra were recorded with the Jasco FP-8300 spectrofluorometer (JASCO GmbH). To measure spectra in the dark samples were handled under a red safety light.

### Spectroscopic determination of the flavin chromophore

For chromophore determination, LreA was isolated from *A*. *alternata* using a pull-down assay and from *E*. *coli* using affinity chromatography. The isolation and determination of the chromophore was carried out according to Faeder and Siegel (43). FAD was used as the flavine-adenine-dinucleotide disodium salt. FMN was used as riboflavin 5′-monophosphate sodium salt hydrate. All steps were carried out under red safety light.

### HPLC analysis

The chromophore from LreA was isolated as described by Faeder and Siegel (43). FAD and FMN chromatographic standards were treated the same. Reverse phase high-performance liquid chromatography was performed on a Vanquish Core HPLC System (Thermo Scientific) equipped with a Hypersil Gold 150 mm × 4.6 mm, 3 μm C18 HPLC column followed by photodiode array detection at a flow rate of 1 ml/min. Buffer and separation conditions were used as described before (66) with the following modifications: prior to sample injection the system was equilibrated for 7 min at 85% solvent A (5 mM NH_4_Ac) and 15% solvent B (100% MeOH). After sample injection, the concentration of B was kept at 15% for 5 min and then raised up to 100% solvent B in 10 min, after which B was kept at 100% for 5 min. Within 3 min the system was returned to equilibration conditions. Relative quantification of FAD and FMN in the extracted protein sample was performed by integrating the eluted peaks between 5.5 min and 7.5 min. FAD was used as flavine-adenine-dinucleotide disodium salt. FMN was used as riboflavin 5′-monophosphate sodium salt hydrate.

### AlphaFold 2, AlphaFill, and protein domain prediction

UniProt database was used to obtain protein sequences (https://www.uniprot.org) (67). We used Expasy Prosite (http://www.prosite.expasy.org) (68), InterPro (https://www.ebi.ac.uk/interpro/) (69), and SMART (http://smart.embl-heidelberg.de) (70) to predict protein domains. Protein sequences were used with AlphaFold 2.3.1 for structural predictions (71, 72). AlphaFill was used to predict the chromophore for the AlphaFold 2 generated models (https://alphafill.eu) (73). Modeling and color coding of AlphaFold 2 predictions, as well as color coding of predicted chromophores by AlphaFill were done with PyMOL 2.4.2.

## Data availability

All data are contained within the manuscript. Strains and plasmids can be obtained upon request.

## Supporting information

This article contains supporting information (27, 64, 74).

## Conflict of interest

The authors declare that they have no conflicts of interest with the contents of this article.
