# Supplementary material for: The LOV-domain blue-light receptor LreA of the fungus Alternaria alternata binds predominantly FAD as chromophore and acts as a light and temperature sensor
Source: J Biol Chem. 2024 Mar 28;300(5):107238. doi: 10.1016/j.jbc.2024.107238 (PMC11061223; doi:10.1016/j.jbc.2024.107238)
Supplement: Tables S1–S3 [file mmc1.pdf]

Supporting information

The LOV-domain blue-light receptor LreA of the fungus *Alternaria alternata* binds predominantly FAD as chromophore and acts as light and temperature sensor

Lars Schuhmacher[1], Steffen Heck[1], Michael Pitz[1], Elena Mathey[1], Tilman Lamparter[2], Alexander Blumhofer[1], Kai Leister[1] and Reinhard Fischer[1*]

running head: light and temperature sensing in Alternaria

Address: [1]Karlsruhe Institute of Technology (KIT) - South Campus
Institute for Applied Biosciences
Dept. of Microbiology
Fritz-Haber-Weg 4
D-76131 Karlsruhe, Germany
Phone: +49-721-6084-4630
Fax: +49-721-6084-4509
E-mail: reinhard.fischer@KIT.edu
Homepage: www.iab.kit.edu

[2]Karlsruhe Institute of Technology (KIT) - South Campus
Joseph Kölreuter Institute for Plant Research
Fritz-Haber-Weg 4
D-76131 Karlsruhe, Germany

* Corresponding author

Table 1: Strains used in this study.

Strain	Organism	Genotype	Reference
ATCC® 66981™	*A. alternata*	wild type	Virginia Bioinform. Inst. (Blacksburg, USA); Lawrence Lab
sOI1	*A. alternata*	Δ*fphA* in *A. alternata* wild type (528 bp deleted by CRISPR/Cas9 mediated mutagenesis)	(27)
sOI3	*A. alternata*	Δ*lreA* in *A. alternata* wild type (3398 bp deleted by CRISPR/Cas9 mediated mutagenesis)	
sHS1	*A. alternata*	*A. alternata* sOI3 complemented with $lreA_{C421S}$ with native promoter and terminator (ectopic integration)	This study
sJJG01	*A. alternata*	Δ*lreA* in *A. alternata s*OI1 (Δ*fphA*) (deleted by CRISPR/Cas9 mediated mutagenesis)	
sLS2	*A. alternata*	*A. alternata* sOI3 complemented with *lreA* with N-terminal 3x *HA-tag* and *gpdA* promoter*; gpdA*(p)::*ha::lreA* (ectopic integration)	
Top10	*E. coli*	F- *mcrA* Δ(*mrr-hsd*RMS-*mcr*BC), Φ80*lacZ*Δ*M15* Δ*lac*X74, *rec*A1, *araD139*Δ (*araleu*)7697, *gal*U, *gal*K, *rps*L (Str^R) *end*A1, *nup*G	Invitrogen, Leek, NL
BL21 DE3	*E. coli*	F- *omp*T *hsd*SB (rB–, mB–) *gal dcm* (DE3)	Novagen, Darmstadt, Germany

Table 2: Plasmids used in this study.

Name	Description	Reference
pHS1	*tet*(p)::*lreA*::*strep-tag*; *Amp*^{R}; *lreA* protein coding sequence (in pASK-IBA3plus)	This study
pHS2	*tet*(p)::*lreA*$_{C421S}$::*strep-tag*; *Amp*^{R}; *lreA* protein coding sequence; mutagenesis of cysteine 421 to serine (in pASK-IBA3plus)	
pHS13	*lreA*$_{C421S}$ including 1 kb up- and downstream of *lreA* wild type gene; *Amp*^{R} (in pJET1.2)	
pHS14	*hph*; *Amp*^{R} (in pJET 1.2)	
pLS32	*alcA*(p) exchanged with 1 kb *gpdA*(p) sequence from *A. alternata* in pSM14 vector; *pyr-4*; *Amp*^{R}	
pLS38	*gpdA*(p)::*HA-tag*::*lreA*; *pyr-4*; *Amp*^{R}; *lreA* ORF sequence including 1,2 kb downstream (in pLS32)	
pJJG1	*tef1*(p)::*spcas9*::*tef1*(t); *gpdA*(p)::*cluster-lreA-sgRNA-casette1*::*trpC*(t); *gpdA*(p)::*cluster-lreA-sgRNA-casette2*::*trpC*(t); *hph*; *Amp*^{R}; *AMA1* (in pFC332)	
pFC332	*tef1(p)::cas9::tef1(t); gpdA(p)::gpdA(t); hph; ampR; AMA1*	Uffe H. Mortensen, Technical University of Denmark
pFC334	*tef1(p)::cas9::tef1(t); gpdA(p)::sgRNA-AnyA::gpdA(t); Afpyr4; ampR; AMA1*	Uffe H. Mortensen, Technical University of Denmark
pAK4	pJET1.2 blunt + *trpC*(p)*::sgRNA-AnyA::trpC*(t)	(64)
pJET 1.2 blunt	Cloning Vector, Component of CloneJET PCR Cloning Kit	Thermo Fisher Scientific, USA
pSM14	*GFP* of pMCB17apx replaced with 3× *HA* between *Kpn*I and *Asc*I restriction sites	(74)

Table 3: Oligonucleotides used in this study.

Name	Sequence (5' → 3')	Function
AaltPgpdA_efi_fw	TTGTAAAACGACGGCCAGTGAATTCGCTTGAGCTCAAGCTGTAGC	For amplifying and cloning the 1 kb *A. alternata gpdA* promoter sequence (plasmid pLS32)
gpdA + ATG in efi HATag r	AACATCGTATGGGTACATGGTACCGGATGAACTGAGTGTAGTGCTG	
LreA ORF AscI (no ATG) f_2	CGTTCCAGATTACGCTGGCGCGCCAAATGGCTATCCATATCCCACC	For amplifying and cloning the open reading frame including 1.2 kb downstream of the *lreA* gene (plasmid pLS38)
LreA 1,2kb do OV pSM14 r	TCGACTCTAGAGGATCCTTAATTAACCCCAATCTAGTTGTAATCTGC	
AaH2B_RT_f	CACCAAGACCAGGAAGGAGA	Amplification of *h2b* gene for RT-qPCR
AaH2B_RT_r	AGTTGAGAATGCTCATGGCG	
AaccgA_RT_74bp_f	CAACAACCCCAACGAGGG	Amplification of *ccgA* gene for RT-qPCR
AaccgA_RT_74bp_r	TCTCGGAGACGTAGTTGGC	
AacatA_RT_108bp_f	AGCTGCCAATTAACAAGCCT	Amplification of *catA* gene for RT-qPCR
AacatA_RT_108bp_r	GGAAACGGTTAGGCCAGTAG	
AacatB_RT_97bp_f	TACATCCGCCATCCAGTTTG	Amplification of *catB* gene for RT-qPCR
AacatB_RT_97bp_r	AGCAGTTGAGTTGGAGTTGG	
AafrqA_RT_114bp_f	GAAAACTCGCAGTATGCCCA	Amplification of *frqA* gene for RT-qPCR
AafrqA_RT_114bp_r	CAGGTCGTCGATAACGCTTC	
AaHSF1_RT_f	TGCGAATATGAACCTTGCGG	Amplification of *hsf8* gene for RT-qPCR
AaHSF1_RT_r	TTGTGAGCATCTTGCATGGG	
AaHSP60_RT_f	AGAAGATCTCCGCAGTCCAG	Amplification of *hsp60* gene for RT-qPCR
AaHSP60_RT_r	CAAGAGCCTCACCGTCAATG	
AaHSP70_RT_f	TGGTTGGACGACAACCAGA	Amplification of *hsp70* gene for RT-qPCR
AaHSP70_RT_r	CCAGCTCCGTAGAACTTCATC	
AaHSP90_RT_f	GGTATCCATGAGGACTCGCA	Amplification of *hsp90* gene for RT-qPCR
AaHSP90_RT_r	CGTAGTCGGTAAGGGAGGTC	
AaHSP104_RT_106bp_f	CGTGTCACAAGAGAATCGCA	

AaHSP104_RT_106bp_r	ATGCCTCATCCTTCTCTCGG	Amplification of *hsp104* gene for RT-qPCR
LreA_C421S_fw	CGGAATTCCCGCTTCTTG	For site directed mutagenesis to change cysteine 421 to serine in *lreA* gene (pHS2, pHS13)
LreA_C421S_rv	TCCAAGAATCATATGTCTTGTGTAG	
pJET_LB_LreA_6kb_ fw	GGCTCGAGTTTTTCAGCAAGATGCGCTCGAATAGATGATATCCC	Amplification of *lreA*$_{C421S}$ open reading frame containing 1 kb up- and downstream for cloning into pJET1.2 (pHS13)
pJET_RB_LreA_6kb_ rv	GTAGGAGATCTTCTAGAAAGATGGCCCCCAATCTAGTTGTA	
pASK_iba3+_LB_ LreA_cds_fw	GTGAAATGAATAGTTCGACAAAAATCTAGAAATAATTTTGTTTAACTTTAAGAAGGAGATATACATATGAATGGCTATCCATATCCCAC	Amplification of *lreA*/*lreA*$_{C421S}$ coding sequence for cloning into pASK-iba3plus (pHS1, pHS2)
pASK_iba3+_RB_ LreA_cds_rv	GCTCCAAGCGCTGAGACCATGGGCGTTGATGGAGCCATTGCCGA	
Protospacer lreA 1 fw	GTC CGT GAG GAC GAA ACG AGT AAG CTC GTC GCG CAC CAC CAC CGT GCC CAG TTT TAG AGC TAG AAA TAG CAA GTT AAA	Amplification of first protospacer sequence for CRISPR/Cas9 plasmid (pJJG1)
HH lreA 1 rev	GAC GAG CTT ACT CGT TTC GTC CTC ACG GAC TCA TCA GGC GCA CCG GTG ATG TCT GCT CAA GCG	
Protospacer lreA 2 fw	GTC CGT GAG GAC GAA ACG AGT AAG CTC GTC AGC TAC GAC AAA TGG AAA AGG TTT TAG AGC TAG AAA TAG CAA GTT AAA	Amplification of second protospacer sequence for CRISPR/Cas9 plasmid (pJJG1)
HH lreA 2 rev mit PtrpC	GAC GAG CTT ACT CGT TTC GTC CTC ACG GAC TCA TCA GAG CTA CAT CGA TGC TTG GGT AGA ATA GG	
